# Suicide awareness homophily in adolescent peer support networks: A Swiss cross-sectional social network analysis

**DOI:** 10.1016/j.pmedr.2024.102747

**Published:** 2024-04-26

**Authors:** Stéphanie Baggio, Marlène Sapin, Neslie Nsingi, Abbas Kanani, Raphaël Thelin

**Affiliations:** a Institute of Primary Health Care (BIHAM), University of Bern, Mittelstrasse 43, 3012 Bern, Switzerland; b Laboratory of Population Health (#PopHealthLab), University of Fribourg, Route des Arsenaux 41, 1700 Fribourg, Switzerland; c Swiss Center of Expertise in Social Sciences (FORS), University of Lausanne, Geopolis Building, 1015 Lausanne, Switzerland; d Department of Community Health Sciences, Max Rady College of Medicine, Rady Faculty of Health Sciences, University of Manitoba, 750 Bannatyne Ave, Winnipeg, MB R3E 0W2, Canada,; e Association Stop Suicide, rue des Savoises 15, 1205 Geneva, Switzerland

**Keywords:** Adolescence, Homophily, Network, Prevention, Suicide awareness, Support

## Abstract

**Objective:**

Suicide awareness, encompassing knowledge, attitudes, and behaviors related to suicide, plays a critical role in primary suicide prevention, especially among adolescents. However, little is known about how perceived suicide awareness is apparent in peer support networks in this population. This study examined the presence of suicide awareness homophily in adolescent peer support networks. We also explored other patterns of homophily and identified factors associated with the in-degree popularity of adolescents.

**Methods:**

We used baseline data from a non-randomized, cluster-controlled trial assessing the effectiveness of a universal suicide prevention intervention in Swiss secondary schools (n = 194). We assessed perceived suicide awareness, support networks (including in-degree popularity, i.e., receiving a high number of nominations as a supportive peer), and other covariates. Data were analyzed using social network analyses.

**Results:**

We found evidence of suicide awareness homophily in peer support networks, where adolescents with high suicide awareness were more likely to connect with peers having high suicide awareness (p < .001). The same applied to those with low suicide awareness (p < .001). Age also emerged as a significant homophily factor. Girls (p = .024) and adolescents with high instrumental social support (p = .008) were more likely to be popular in peer support networks.

**Conclusions:**

This study highlighted the homophily of suicide awareness in peer support networks and the need to focus on strengthening peer support networks and promoting suicide awareness in adolescents, particularly for those with low suicide awareness. Future suicide prevention programs, including peer-led interventions, should consider these findings to better target vulnerable subgroups and reduce suicide-related disparities.

## Highlights

• Little is known about suicide awareness homophily in adolescent peer support networks.
• Adolescents are likely to connect with peers sharing similar suicide awareness levels.
• Fostering suicide awareness is crucial for effective suicide prevention programs.
• Relying on suicide awareness homophily can help building community support.
• It can also help reinforcing support networks through the influence of popular adolescents.

## 1 Introduction

### 1.1 Suicide awareness

The concept of suicide awareness is a multidimensional construct, encompassing elements of knowledge (risk factors and warning signs of suicide) and attitudes (myths and preconceived ideas about suicide), often referred to as “suicide literacy” (Ludwig et al., 2022). Additionally, it includes responses and behaviors related to seeking help for oneself and others in case of suicidal thoughts and behaviors (Cusimano and Sameem, 2011). Suicide awareness also encompasses “perceived suicide awareness”, which involves how people feel about talking about suicide and seeking help. More specifically, perceived suicide awareness deals with one’s perceived knowledge, confidence, and willingness to talk about suicide and seek help, which could be considered as a new dimension of suicide awareness (Baggio et al., 2024). As suicide awareness encompasses knowledge, attitudes, and behaviors (Cusimano and Sameem, 2011, Ludwig et al., 2022) as well as perceptions (Baggio et al., 2024) related to suicide, it is also important for suicide prevention. Indeed, improving suicide awareness can empower individuals to recognize signs of distress, seek help when needed, and advocate for improved mental health resources and support systems within their communities. Therefore, suicide awareness can help promote help-seeking behavior and build community support. In this study, we focused on perceived suicide awareness. However, there is a dearth of studies examining associations of suicide awareness with essential variables, such as support networks.

### 1.2 Support networks, mental health, and suicide awareness

Decades of research and theory have highlighted the health-promoting effects of social support (Berkman et al., 2000, Schuster et al., 1990, Thoits, 2011). Personal support networks have been consistently reported as protective factors for mental health (Borowsky et al., 2001, Pisani et al., 2013, Resnick et al., 1997). To date, few studies have examined how suicide awareness, which is important for suicide prevention, is associated with support networks that have health-promoting effects. A study focusing on Japanese adults reported that a high suicide literacy was associated with a greater social support (Nakamura et al., 2021). A systematic review showed that a low social support was a barrier to seeking professional help for suicidal ideation (Han et al., 2018). Suicide literacy positively impacts help-seeking behaviors (Žilinskas and Lesinskienė, 2023) and access to professional help reduces the risk of suicide and adult psychiatric disorders (Michelmore and Hindley, 2012). Therefore, exploring the relationship between suicide awareness and social networks may be a promising avenue for suicide prevention. To our knowledge, advanced social network analyses have not been used to examine the structure of support networks in relation to suicide awareness.

### 1.3 Homophily and in-degree popularity in peer support networks

Previous research has shown that social networks and health behaviors are intertwined (Daw et al., 2015). An important feature of social networks is homophily, which corresponds to the predominance of within-category ties (McPherson et al., 2001). It means that contact (e.g., friendship, support relationship) is more likely to occur between similar people than between dissimilar people. Patterns of homophily have been observed for a wide range of factors, including sociodemographics (Goodreau et al., 2009, McPherson et al., 2001), physical health (Copeland et al., 2023, Schaefer and Simpkins, 2014), and mental health (Baggio et al., 2017, Schaefer et al., 2011). Previous studies have shown that homophily based on health-related characteristics influences health behaviors (Flatt et al., 2012). For example, smokers are more likely to connect with other smokers, and smoking cessation may spread through the network (Christakis and Fowler, 2008). However, few studies have focused on homophily patterns in peer support networks, especially in adolescents (Mamas et al., 2020, Wang et al., 2021).

In-degree popularity refers to receiving a high number of friendship nominations in a network (Kadushin, 2012). It corresponds to in-degree (number of incoming connections) by opposition to out-degree (number of outgoing connections). Friendship popularity is associated with having more supportive friendships (Litwack et al., 2012). Leveraging the influence of popular individuals within peer support networks can be a valuable strategy for promoting mental health support and suicide prevention efforts. However, popularity in adolescent peer support networks has been understudied.

### 1.4 Objectives

To fill in previous research gaps, this study examined how perceived suicide awareness is related to peer support networks. We hypothesized that adolescents with high suicide awareness would be more likely to associate with peers who also have high suicide awareness, while adolescents with low suicide awareness would be more likely to associate with peers who have low suicide awareness. The study had two secondary objectives: 1) to identify other patterns of homophily in peer support networks, including sociodemographic, i.e., age, sex, and parental level of education; and psychological variables. i.e., coping skills, psychological distress, and suicidal ideation), and 2) to identify factors associated with in-degree popularity in peer support networks to identify key adolescents in the peer support network.

## 2 Methods

### 2.1 Design and setting

Data used in this study were collected during the baseline assessment of a non-randomized, cluster-controlled trial designed to test the effectiveness of a universal suicide prevention intervention (Baggio et al., 2019, Baggio et al., 2022). The study took place in December 2019 and October 2020 in twelve classes of a secondary school of a canton of the French-speaking part of Switzerland, Neuchâtel. The cantonal ethics committee considered the study to be outside the scope of Swiss legislation and issued a waiver (no. 2019–00295). The adolescents gave written informed consent before study participation.

### 2.2 Participants

Inclusion criteria were 1) age 14 or older, 2) ability to communicate in French, and 3) enrollment at the Neuchâtel study site. The main trial also took place in a school located in the canton of Geneva, but we excluded participants because support networks could not be adequately assessed in this school. Out of 234 eligible adolescents, 214 agreed to participate (response rate = 92 %). We excluded 20 participants due to missing information on the suicide awareness scale and other variables, leaving 194 participants in the final sample (91 % of respondents). Students from the twelve classes were regrouped for specific courses, resulting in six “class groups” of students who knew each other.

### 2.3 Procedures

Baseline data were collected prior to the universal suicide prevention intervention conducted by the association *Stop Suicide* (https://stopsuicide.ch). Participants received information about the study and provided informed consent. They then completed the baseline assessment (paper-and-pencil questionnaire of approximately 20 min) in the classroom on the same day of the intervention, before the intervention. They received no compensation for their participation in the study. A psychologist was available during and after the intervention in case any student needed counseling or referral.

### 2.4 Measures

#### 2.4.1 Perceived Suicide Awareness Scale (PSAS-9)

The PSAS-9 is a nine-item scale that assesses perceived suicide awareness. It includes three items on perceived knowledge about suicide and help-seeking resources (e.g., “I have the knowledge to talk about suicide with others”), three items on willingness to talk about suicide and to seek for help (e.g., “I would be willing to seek help for suicidal thoughts”), and three items on confidence to talk about suicide and seek for help (e.g., “I would feel confident when it comes to recognizing warning signs for suicide in others”) (Baggio et al., 2024). Items are rated on a five-point scale and a total score is derived, ranging from 0 (low perceived suicide awareness) to 36 (high perceived suicide awareness). The internal consistency of the scale was α = 0.75 (α = 0.78 in the validation study, see Baggio et al., 2024). A binary variable was created for network analyses, with a cut-point in the middle of the 36-point scale (≤ 18 vs. > 18).

#### 2.4.2 Support networks

Participants reported the name of their classmates (only in the class group) with whom they felt comfortable talking about problems, with no maximum number. We computed in-degrees (number of incoming connections) and out-degrees (number of outgoing connections). For analyses, we used a binary indicator of in-degree popularity, with in-degree popularity defined as being selected by at least two other participants as a supportive peer (Jiao et al., 2017).

#### 2.4.3 Coping skills

We used the French version of the COPE inventory to assess three relevant coping skills: Planning, seeking of instrumental social support, and seeking of emotional social support (Carver et al., 1989). We calculated a mean score for each four-item subscale (ranging from 0 = strongly disagree to 3 = strongly agree). The internal consistency of the scales were α = 0.71 for planning, α = 0.84 for instrumental social support, and α = 0.84 for emotional social support (respectively α = 0.80, 0.75, and 0.85 in the validation study, Carver et al., 1989). We used binary variables for network analyses, with a cut-point in the middle of the scale (≤ 1.5 vs. > 1.5).

#### 2.4.4 Psychological distress

We assessed psychological distress over the previous four weeks using the Kessler Psychological Distress Scale (K-6) (Arnaud et al., 2010, Kessler et al., 2002). The K-6 has six items and is scored on a five-point scale. We calculated a total score ranging from 0 to 24. The internal consistency of the scale was α = 0.84 (α = 0.89 in the validation study, Kessler et al., 2002). For analyses of the homophily patterns, we used a cut-off of 13 or more to indicate moderate or severe psychological distress, as suggested by Kessler et al. (2002) who found a total classification accuracy of 0.92 in the validation study).

#### 2.4.5 Suicidal ideation

We assessed lifetime presence or absence of suicidal ideation by endorsing one of the two items “wish to be dead” and “nonspecific suicidal thoughts” from the French version of the Columbia Suicide Severity Rating Scale (C-SSRS) (Posner et al., 2011).

#### 2.4.6 Sociodemographics

Sociodemographic variables included age (14 vs. 15–16 years old, as only 5 participants were 16), gender, and parental education level (primary or secondary vs. tertiary).

### 2.5 Statistical analyses

#### 2.5.1 Preliminary analyses

We first performed descriptive statistics for all study variables. We then tested the association between the PSAS-9 and characteristics using mixed-effects linear regression models. We ran bivariable and multivariable models with participants nested into class groups.

#### 2.5.2 Social network analyses

We analyzed support relationships using the framework of the Exponential Random Graph Model (ERGM) (Morris et al., 2008). ERGM allows for testing the effect of individual-level variables on ties, while also accounting for tie-interdependent structures. It accounts for reciprocity (“I am friend with my friends”), transitivity (“My friends’ friends are my friends”), and homophily. ERGMs are applied to closed networks, such as an entire class or a school. In our case, networks corresponded to each class group (k = 6).

We first computed network density (i.e., the sum of the ties divided by the number of possible ties), reciprocity (i.e., the proportion of symmetric dyads), and transitivity (i.e., the proportion of triads). To answer our primary and first secondary research questions, we then computed ERGMs to test for the presence of homophily in the network. We computed separate models for the PSAS-9 and each covariate, controlling for reciprocity and transitivity. We then compute a multivariable model including all covariables. Parameters were calculated using Markov chain Monte Carlo maximum likelihood estimation (Robins et al., 2007).

Since ERGM does not allow missing values, we imputed missing values for the covariables (parental level of education: 5 missing values, coping: 2 missing values, psychological distress: 2 missing values, and suicidal ideation: 3 missing values). We imputed missing values using multivariate imputation by chained equations, imputing a single value, as ERGM does not support multiple imputation. We used the integrated approach to compute network density, reciprocity, and transitivity. The integrated approach merges all networks altogether and imposes structural zeros between participants from different networks (Tolochko and Boomgaarden, 2024). As the number of class groups was too small, we could not use a fully integrated approach in ERGM (no convergence). In addition, because the number of support ties was too small to analyze classes separately (lack of power), we used a single ERGM to analyze class groups altogether.

To answer our second secondary research question, we tested which variables predicted in-degree popularity using a logistic regression model, with all covariates used as factors, with bootstrapped standard errors (n = 1000) to account for the lack of independence between observations. We ran a sensitivity analysis using an alternative cut-off score for the PSAS-9 (≥22, the mean score of the sample). Results were similar to those reported in the Results section. We performed statistical analyses with R version 4.3.1 (packages mice version 3.16.0 and mlergm 0.8).

## 3 Results

Descriptive statistics are shown in Table 1. The mean age of participants was 14.4 ± 0.5 years, 54.1 % were girls. Associations between the PSAS-9 and covariables are shown in Table 2. In bivariable and multivariable associations, the PSAS-9 was significantly associated with two coping skills, planning and instrumental social support (p < .043).

**Table 1:** Descriptive statistics among 194 adolescents, 2019–2020, Switzerland.

Variables		Mean (sd, range) /Percentage (n)
Age^1^		14.4 (0.5, 14–16)
	Age 14^2^	61.9 (120)
	Age 15 or 16^2^	38.1 (74)
Gender^2^		
	Girls	54.1 (102)
	Boys	45.9 (89)
Parental level of education^2^		
	Primary or secondary	64.4 (125)
	Tertiary	35.6 (69)
Perceived suicide awareness scale (PSAS-9) (0–36)^1^		22.0 (5.5, 3–35)
	PSAS-9 ≤ 18^2^	23.7 (46)
	PSAS-9 > 18^2^	76.3 (148)
Coping skills: planning (0–3)^1^		1.5 (0.7, 0–3)
	Planning ≤ 1.5^2^	55.7 (108)
	Planning > 1.5^2^	44.3 (86)
Coping skills: instrumental social support (0–3)^1^		1.2 (0.8, 0–3)
	Instrumental social support ≤ 1.5^2^	73.2 (142)
	Instrumental social support > 1.5^2^	26.8 (52)
Coping skills: emotional social support (0–3)^1^		1.6 (0.8, 0–3)
	Emotional social support ≤ 1.5^2^	51.6 (100)
	Emotional social support > 1.5^2^	48.5 (94)
Psychological distress^1^		8.2 (5.3, 0–23)
	Psychological distress ≤ 12^2^	76.8 (149)
	Psychological distress > 12^2^	23.2 (45)
Lifetime suicidal ideation^2^		
	No	54.1 (105)
	Yes	45.9 (89)
Out-degree		
	Median (interquartile range)	1 (2, 0–8)
	0^2^	46.4 (90)
	1^2^	20.6 (40)
	2^2^	16.5 (32)
	3 or more^2^	16.5 (32)
In-degree (popularity)		
	Median (interquartile range)	1 (2, 0–4)
	0^2^	37.1 (72)
	1^2^	32.0 (62)
	2^2^	17.5 (34)
	3 or more^2^	13.4 (26)

1 Means and standard deviations are given. ^2^ Percentages and n are given.

**Table 2:** Associations between the PSAS-9 and covariables among 194 adolescents, 2019–2020, Switzerland.

Variables		Bivariable models		Multivariable model	
		b	p	b	p
Age					
	Age 14	Ref.	−	Ref.	−
	Age 15 or 16	−0.23	.781	−0.03	.966
Gender					
	Girls	Ref.	−	Ref.	−
	Boys	−1.11	.161	−0.89	.255
Parental level of education					
	Primary or secondary	Ref.	−	Ref.	−
	Tertiary	0.62	.451	0.34	.649
Coping skills: planning					
	Planning ≤ 1.5	Ref.	−	Ref.	−
	Planning > 1.5	1.31	.100	1.22	.043
Coping skills: instrumental social support					
	Instrumental social support ≤ 1.5	Ref.	−	Ref.	−
	Instrumental social support > 1.5	2.94	.001	1.47	.008
Coping skills: emotional social support					
	Emotional social support ≤ 1.5	Ref.	−	Ref.	−
	Emotional social support > 1.5	2.90	<.001	0.57	.290
Psychological distress					
	Psychological distress ≤ 12	Ref.	−	Ref.	−
	Psychological distress > 12	−1.52	.097	−1.36	.177
Lifetime suicidal ideation					
	No	Ref.	−	Ref.	−
	Yes	−1.03	.195	0.11	.900

PSAS-9: nine-item Perceived Suicide Awareness Scale.

Mixed-effect linear regressions were computed. Non-standardized b estimates and p-values are reported.

**Fig. 1:**
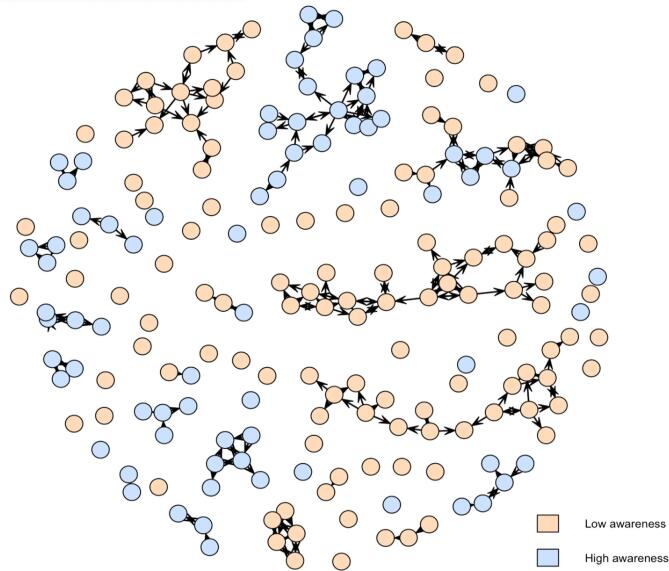
Integrated support network according to suicide awareness score among 194 adolescents, 2019–2020, Switzerland. Each node represents one adolescent. Arrows show the direction of the support relationship. Low awareness corresponds to a score of the 9-item Perceived Suicide Awareness Scale (PSAS-9) < 18, high awareness to a score ≥ 18. k = 6 class groups.

**Fig. 2:**
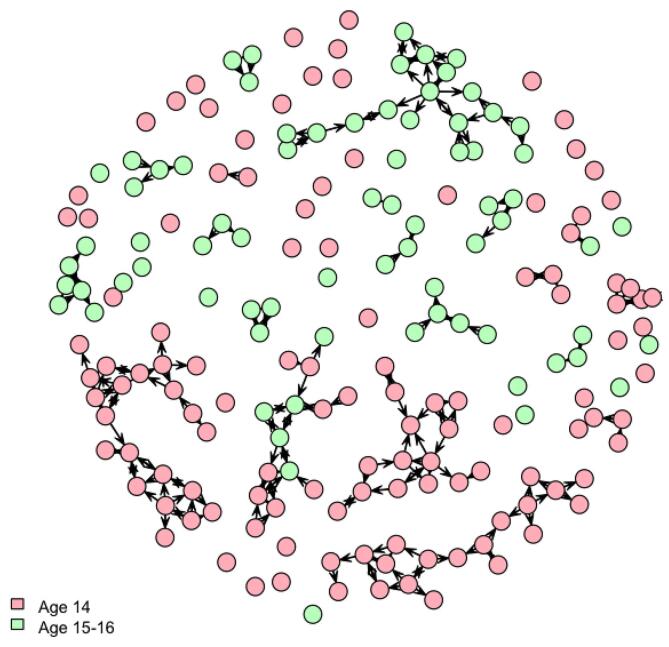
Integrated support network according to age among 194 adolescents, 2019–2020, Switzerland. Each node represents one adolescent. Arrows show the direction of the support relationship. k = 6 class groups.

The network density was 0.6 %, meaning that support relationships were very sparse in the network. Reciprocity was 99.4 %, and transitivity was 38.0 %, highlighting reciprocity within support relationships and the presence of subgroups in sparse connections. The results of the ERGMs are shown in Table 3. There were homophily patterns for two variables: the PSAS-9 and age. Participants with low suicide awareness were more likely to connect for support relationships (b = 0.97, p < .001), as were participants with high suicide awareness (b = 0.52, p < .001). The support network, according to participants’ PSAS-9 score, is shown in Fig. 1. Participants aged 14 were more likely to seek support from peers of their age (b = 1.86, p < .001), as were participants aged 15 or 16 (b = 2.11, p < .001). The support network by age is presented in Fig. 2. Supplementary material shows support networks by PSAS-9 score and age separately for each class group. The other covariates, including sex, parental level of education, coping skills, psychological distress, and suicidal ideation did not display significant homophily patterns (p ≥ .255).

**Table 3:** Results of the ERGM testing homophily patterns in support relationships among 194 adolescents, 2019–2020, Switzerland.

Variables		Model A		Model B	
		b	p	b	p
PSAS-9					
	< 18	0.95	<.001	0.97	<.001
	≥ 18	0.53	<.001	0.52	<.001
Age					
	Age 14	1.88	<.001	1.86	<.001
	Age 15 or 16	2.10	<.001	2.11	<.001
Gender					
	Girls	0.06	.639	0.10	.406
	Boys	0.09	.472	0.04	.772
Parental level of education					
	Primary or secondary	−0.11	.320	−0.10	.377
	Tertiary	−0.21	.270	−0.23	.255
Coping skills: planning					
	Planning ≤ 1.5	0.17	.224	0.09	.535
	Planning > 1.5	0.08	.460	0.05	.662
Coping skills: instrumental social support					
	Instrumental social support ≤ 1.5	0.05	.645	0.04	.747
	Instrumental social support > 1.5	−0.05	.766	−0.05	.781
Coping skills: emotional social support					
	Emotional social support ≤ 1.5	0.14	.288	0.11	.418
	Emotional social support > 1.5	−0.01	.911	−0.02	.867
Psychological distress					
	Psychological distress ≤ 12	0.06	.561	0.03	.833
	Psychological distress > 12	−0.06	.849	−0.12	.697
Lifetime suicidal ideation					
	No	0.12	.289	0.13	.272
	Yes	−0.01	.972	−0.02	.887

ERGM: exponential random graph model; PSAS-9: 9-item Perceived Suicide Awareness Scale.

Results of ERGM (outcome: presence of a support relationship) for each covariate controlling for reciprocity and transitivity (model A) and for multivariable model including all covariables, reciprocity, and transitivity (model B) are reported.

Results of the logistic regression model predicting in-degree popularity are shown in Table 4. Bivariable models showed significant effects of gender (odds-ratio [OR] = 0.34, p = .002), instrumental social support (OR = 3.18, p = .001), and emotional social support (OR = 2.17, p = .020). Girls and participants with high levels of coping skills were more likely to be popular in providing support. In the multivariable model, gender (OR = 0.41, p = .024) and instrumental social support (OR = 3.15, p = .008) remained significant.

**Table 4:** Associations between in-degree popularity and covariables among 194 adolescents, 2019–2020, Switzerland.

Variables		Bivariable models		Multivariable model	
		OR	p	OR	p
Age					
	Age 14	Ref.	−	Ref.	−
	Age 15 or 16	0.91	.787	0.96	.913
Gender					
	Girls	Ref.	−	Ref.	−
	Boys	0.34	.002	0.41	.024
Parental level of education					
	Primary or secondary	Ref.	−	Ref.	−
	Tertiary	1.62	.146	1.66	.199
PSAS-9					
	PSAS-9 ≤ 18	Ref.	−	Ref.	−
	PSAS-9 > 18	0.90	.780	0.55	.236
Coping skills: planning					
	Planning ≤ 1.5	Ref.	−	Ref.	−
	Planning > 1.5	0.86	.634	0.84	.680
Coping skills: instrumental social support					
	Instrumental social support ≤ 1.5	Ref.	−	Ref.	−
	Instrumental social support > 1.5	3.18	.001	3.15	.009
Coping skills: emotional social support					
	Emotional social support ≤ 1.5	Ref.	−	Ref.	−
	Emotional social support > 1.5	2.17	.020	1.63	.249
Psychological distress					
	Psychological distress ≤ 12	Ref.	−	Ref.	−
	Psychological distress > 12	1.50	.294	1.00	.992
Lifetime suicidal ideation					
	No	Ref.	−	Ref.	−
	Yes	1.54	.158	1.40	.465

PSAS-9: nine-item Perceived Suicide Awareness Scale.

Logistic regressions with bootstrapped standard errors (n = 1000) were computed. Odds-ratios (OR) and p-values are reported.

## 4 Discussion

### 4.1 Primary objective: Suicide awareness homophily in peer support networks

The findings confirmed our hypothesis about the presence of suicide awareness homophily in peer support networks. They are consistent with previous research suggesting homophily patterns of health behaviors (Baggio et al., 2017, Copeland et al., 2023, Schaefer et al., 2011, Schaefer and Simpkins, 2014). Importantly, suicide awareness homophily exists independently of other adolescent characteristics and was not confounded by any other factors. Adolescents with low suicide awareness are less likely to know where to seek help when they have suicidal thoughts and they are less likely to receive useful help when they turn to peers for social support, as supportive peers also have a low suicide awareness. The mechanism of homophily is a well-documented source of social inequalities, which can also lead to health inequalities (Klärner et al., 2022). Suicide awareness homophily may also drive health inequalities, with the most vulnerable being less able to receive adequate support from their peer network.

This finding may have implications for suicide prevention programs, questioning programs that focus on peer-led interventions. For example, in peer gatekeeper programs, some adolescents (the gatekeepers) are trained in suicide prevention and to provide peer-to-peer assistance. The evidence on peer-led interventions is mixed. A recent systematic review stated that no firm conclusions could be drawn about the effectiveness of peer-led mental health interventions and that studies should better target the children most likely to benefit from it (King and Fazel, 2021). Another systematic review concluded that school-based peer-led educational interventions were effective in improving health (Dodd et al., 2022), but in this review, only one study had a positive effect on help-seeking behavior for mental health problems (Wyman et al., 2010). As suicide awareness is shaped by peer support networks, we suggest that future studies of peer-led interventions include social support measures to better test the effectiveness of the intervention.

### 4.2 First secondary objective: Other types of homophily in peer support networks

We identified age as a pattern of homophily in peer support networks. Adolescents aged 14 were more likely to seek support from peers of the same age, and the same was true for adolescents aged 15–16. Age homophily in peer support networks during adolescence may facilitate the formation of meaningful connections and support systems among individuals who share similar developmental experiences. Age also serves as a basis for social identity during adolescence, as adolescents share a stronger sense of belonging and connection with peers of the same age, facilitating the formation of supportive relationships.

Other factors, including sociodemographics (gender and parental level of education) and psychological variables (coping skills, psychological distress, and suicidal ideation), were not homophily factors in peer support networks. This differed from two recent studies that found patterns of homophily in support networks by gender (Mamas et al., 2020, Wang et al., 2021). To our knowledge, few studies have focused on patterns of homophily using ERGM in adolescent support networks, and further research is needed to achieve a better understanding of these network features.

### 4.3 Second secondary objective: Factors predicting in-degree popularity in peer support networks

Popular peers, participants nominated by at least two other participants as supportive peers, were more likely to be girls and to have high instrumental social support. Previous research has shown that there is a greater intimacy among girls, because of an emphasis on the importance of social relationships since childhood (Shrum et al., 1988). This may explain why they hold a more popular position in peer support networks. Instrumental social support is a form of coping skill referred to as problem-solving. When problems arise, participants with high instrumental social support try to get advice and emotional support, including from people who have had similar experiences, find out more about the situation, or do something concrete about the problem (Carver et al., 1989). Therefore, participants seemed to seek out classmates susceptible to provide concrete social support when they faced a problem.

In contrast, other covariates were not significantly associated with in-degree popularity. This was the case for suicidal ideation and psychological distress, meaning that the suicide- or distress-related experience had no influence on the selection of a supportive peer. These network characteristics may inform suicide prevention programs, both to build on existing skills and to enhance peer support skills.

### 4.4 Other relevant findings

The overall density of the support network was very low (0.6 %). The median number of support relationships (out-degree) was 1. This finding was in line with those of a previous study reporting a sparse support network in Chinese adolescents (Wang et al., 2021). This means that adolescents reported very few classmates with whom they felt comfortable talking about problems. Because the study focused on closed peer support networks, it did not mean that they had no one to talk to about problems, but it did suggest that the class may not be the best natural environment in which to talk about problems.

Reciprocity was very high (99.4 %), and transitivity was high (38.0 %) in the peer support network. This is also coherent with previous findings suggesting that reciprocity and transitivity are important features in social support (Wang et al., 2021). These results may also inform future research aiming at improving suicide awareness through school-based interventions.

### 4.5 Study strengths and limitations

To our knowledge, this was the first time that homophily patterns and popularity in support networks were tested in relation to suicide awareness and psychological variables, providing a unique opportunity to examine the potential importance of social networks in health inequalities.

However, there are several limitations. First, we relied on a single school, so the results should be interpreted cautiously. Second, we used the class group as the unit of analysis, which may create artificial social groups and oversimplify the complexity of adolescents' social networks. Third, the study had a cross-sectional design, and we could not test for dynamic associations between suicide awareness and social support. The initial trial included a one-month follow-up, a too-short period to detect significant changes in support networks. Thus this study focused on the baseline assessment. Future studies with longer follow-ups are needed to examine how social support and suicide awareness influence each other, as well as how they relate to suicide risk. Another limitation was that we assessed binary ties (presence or absence of support) (McMillan, 2022). Ordinal scales could be used to examine more in-depth peer support networks, using valued ERGMs. However, valued ERGMs can cause computational difficulties (Huang and Butts, 2024). Finally, we could not use the integrated approach to compute the ERGMs, because of the small number of class groups (k = 6) and computational difficulties. Future studies should replicate our findings in larger samples and different schools to confirm the homophily pattern on suicide awareness identified in this study.

## 5 Conclusion

In conclusion, this study highlighted the homophily of suicide awareness in adolescent peer support networks. The cross-sectional design of the study did not allow for the determination of the causal relationship between these two variables, but perceived suicide awareness and peer support networks appeared to be intertwined. This finding is useful to inform future suicide prevention programs, especially those relying on peer-to-peer support, of the need to focus on youth with low suicide awareness.

## 6 Funding statement

The work was supported by Promotion Santé Suisse.

## CRediT authorship contribution statement

**Stéphanie Baggio:** Writing – original draft, Validation, Methodology, Formal analysis, Conceptualization. **Marlène Sapin:** Writing – review & editing, Validation, Conceptualization. **Neslie Nsingi:** Writing – review & editing, Investigation, Data curation. **Abbas Kanani:** Writing – review & editing, Investigation, Data curation, Conceptualization. **Raphaël Thelin:** Writing – review & editing, Project administration, Investigation, Funding acquisition, Data curation, Conceptualization.

## Declaration of competing interest

The authors declare that they have no known competing financial interests or personal relationships that could have appeared to influence the work reported in this paper.

## Data Availability

Data will be made available on request.
