# Supplementary material for: Suicide awareness homophily in adolescent peer support networks: A Swiss cross-sectional social network analysis
Source: Prev Med Rep. 2024 Apr 26;42:102747. doi: 10.1016/j.pmedr.2024.102747 (PMC11067475; doi:10.1016/j.pmedr.2024.102747)
Supplement: Supplementary Data 1 [file mmc1.docx]

**Supplementary material**

Suppl. Figure 1. Support network according to suicide awareness score in adolescents of class group 1, 2019-2020, Switzerland

Each node represents one adolescent. Arrows show the direction of the support relationship.

Low awareness corresponds to a score of the 9-item Perceived Suicide Awareness Scale (PSAS-9) < 18, high awareness to a score ≥ 18.

Suppl. Figure 2. Support network according to age in adolescents of class group 1, 2019-2020, Switzerland

Each node represents one adolescent. Arrows show the direction of the support relationship.

Suppl. Figure 3. Support network according to suicide awareness score in adolescents of class group 2, 2019-2020, Switzerland

Each node represents one adolescent. Arrows show the direction of the support relationship.

Low awareness corresponds to a score of the 9-item Perceived Suicide Awareness Scale (PSAS-9) < 18, high awareness to a score ≥ 18.

Suppl. Figure 4. Support network according to age in adolescents of class group 2, 2019-2020, Switzerland

Each node represents one adolescent. Arrows show the direction of the support relationship.

Suppl. Figure 5. Support network according to suicide awareness score in adolescents of class group 3, 2019-2020, Switzerland

Each node represents one adolescent. Arrows show the direction of the support relationship.

Low awareness corresponds to a score of the 9-item Perceived Suicide Awareness Scale (PSAS-9) < 18, high awareness to a score ≥ 18.

Suppl. Figure 6. Support network according to age in adolescents of class group 3, 2019-2020, Switzerland

Each node represents one adolescent. Arrows show the direction of the support relationship.

Suppl. Figure 7. Support network according to suicide awareness score in adolescents of class group 4, 2019-2020, Switzerland

Each node represents one adolescent. Arrows show the direction of the support relationship.

Low awareness corresponds to a score of the 9-item Perceived Suicide Awareness Scale (PSAS-9) < 18, high awareness to a score ≥ 18.

Suppl. Figure 8. Support network according to age in adolescents of class group 4, 2019-2020, Switzerland

Each node represents one adolescent. Arrows show the direction of the support relationship.

Suppl. Figure 9. Support network according to suicide awareness score in adolescents of class group 5, 2019-2020, Switzerland

Each node represents one adolescent. Arrows show the direction of the support relationship.

Low awareness corresponds to a score of the 9-item Perceived Suicide Awareness Scale (PSAS-9) < 18, high awareness to a score ≥ 18.

Suppl. Figure 10. Support network according to age in adolescents of class group 5, 2019-2020, Switzerland

Each node represents one adolescent. Arrows show the direction of the support relationship.

Suppl. Figure 11. Support network according to suicide awareness score in adolescents of class group 6, 2019-2020, Switzerland

Each node represents one adolescent. Arrows show the direction of the support relationship.

Low awareness corresponds to a score of the 9-item Perceived Suicide Awareness Scale (PSAS-9) < 18, high awareness to a score ≥ 18.

Suppl. Figure 12. Support network according to age in adolescents of class group 6, 2019-2020, Switzerland

Each node represents one adolescent. Arrows show the direction of the support relationship.
